# Epidermal growth factor receptor inhibitors trigger a type I interferon response in human skin

**DOI:** 10.18632/oncotarget.10013

**Published:** 2016-06-14

**Authors:** Daniela Lulli, Maria Luigia Carbone, Saveria Pastore

**Affiliations:** ^1^ Laboratory of Experimental Immunology, IDI-IRCCS, Fondazione Luigi M. Monti, Rome, Italy

**Keywords:** anticancer drug, transcriptome profiling, tumor necrosis factor alpha, chemokine, antiviral innate immunity

## Abstract

The Epidermal Growth Factor Receptor (EGFR) is centrally involved in the regulation of key processes of the epithelia, including cell proliferation, survival, differentiation, and also tumorigenesis. Humanized antibodies and small-molecule inhibitors targeting EGFR were developed to disrupt these functions in cancer cells and are currently used in the treatment of diverse metastatic epithelial cancers. By contrast, these drugs possess significant skin-specific toxic effects, comprising the establishment of a persistent inflammatory milieu. So far, the molecular mechanisms underlying these epiphenomena have been investigated rather poorly. Here we showed that keratinocytes respond to anti-EGFR drugs with the development of a type I interferon molecular signature. Upregulation of the transcription factor IRF1 is early implicated in the enhanced expression of interferon-kappa, leading to persistent activation of STAT1 and further amplification of downstream interferon-induced genes, including anti-viral effectors and chemokines. When anti-EGFR drugs are associated to TNF-α, whose expression is enhanced by the drugs themselves, all these molecular events undergo a dramatic enhancement by synergy mechanisms. Finally, high levels of interferon-kappa can be observed in epidermal keratinocytes and also in leukocytes infiltrating the upper dermis of cetuximab-driven skin lesions. Our data suggest that dysregulated activation of type I interferon innate immunity is implicated in the molecular processes triggered by anti-EGFR drugs and leading to persistent skin inflammation.

## INTRODUCTION

Epidermal growth factor receptor (EGFR) inhibitors are increasingly used in monotherapy or in combination with chemo/radiotherapy for the treatment of diverse metastatic epithelial cancers [1]. Monoclonal antibodies such as cetuximab and panitumumab inhibit activation of the EGFR by partially occluding its extracellular ligand binding region, and by preventing the receptor from adopting the conformation required for dimerization and initiation of signal transduction [2, 3]. In contrast, tyrosine kinase inhibitors such as gefitinib and erlotinib, interfere with the enzymatic activity of the intracellular EGFR tyrosine kinase domain by competing with ATP binding site [4]. Several adverse effects can be severe enough to markedly reduce treatment adherence or lead to discontinuation, the earliest and most common manifesting on the skin [5, 6]. Within weeks from the first administration, EGFR inhibitors typically induce a papulopustular exanthema, histologically characterized by a dense superficial dermal inflammatory infiltrate surrounding hyperkeratotic and ectatic follicular infundibula. The infiltrate is initially formed of clusters of macrophages, dendritic cells and abundant lymphocytes, in the context of a progressive disruption of physical/chemical and antimicrobial epidermal barriers due to keratinocyte dysfunction and/or death [7, 8].

Several years ago we published the first *in vitro* evidence that the EGFR-ligand system has a major impact on the pro-inflammatory functions of normal human keratinocytes. In particular, we showed that pharmacological blockade of EGFR boosts the expression of the monocyte-, dendritic cell- and T cell-directed chemoattractants CCL2 and CCL5, and the T cell-selective CXCL10, whereas it dramatically impaired the expression of GM-CSF and CXCL8 [9–13]. A sound confirmation that the EGFR-driven immunoregulatory function is a keratinocyte-autonomous event was finally provided by two more recent papers from independent laboratories [8, 14]. By performing parallel investigations on biopsies from anti-EGFR drug-treated cancer patients and on mouse models with EGFR ablation in the epidermis, these Authors confirmed up-regulated expression of pro-inflammatory mediators, including the pro-inflammatory cytokine TNF-α, and the chemokines CCL2, CCL5 and CXCL10 [8, 14]. Notably, subcutaneous injections of the type I interferon (IFN) β in multiple sclerosis patients were shown to initiate an inflammatory skin reaction characterized by enhanced expression of these chemokines in keratinocytes and infiltrating leucocytes [15].

Type I IFNs are key innate immune cytokines produced by cells to trigger antiviral, antitumor and immunostimulatory functions [16–18]. In humans, IFN-α, with 13 partially homologous isoforms, and IFN-β1, the product of a single gene, are the best characterized type I IFNs. This class of cytokines also comprises the subtypes IFN-ε, IFN-κ and IFN-ω, whose expression is more cell-restricted. In particular, IFN-κ, initially identified as the keratinocyte-specific type I IFN [19], was found highly expressed also in monocytes and dendritic cells infiltrating chronic inflammatory skin lesions [20]. Repression of constitutive IFN-κ transcription in keratinocytes is the major strategy of innate immune evasion by carcinogenic papillomaviruses [21–23]. All type I IFNs share a ubiquitously expressed heterodimeric receptor, IFN α/β receptor (IFNAR), with IFNAR1 and IFNAR2 chains signalling through two Janus family kinases, Tyk2 and Jak1, and leading to recruitment of STAT1 to receptor-bound STAT2, their phosphorylation and formation of STAT1-STAT2 heterodimers. In the nucleus, these heterodimers associate with the transcription factor IFN Regulatory Factor (IRF) 9 to form the heterotrimeric complex IFN-stimulated gene factor 3, which binds to IFN-stimulated response elements in the promoter of IFN-inducible genes and activates their transcription. Importantly, IFNAR can also signal by inducing the activation and nuclear translocation of phosphorylated STAT1 homodimers, which bind to IFN-γ-activated sequences in the promoters of IFN-γ-induced genes. Eventually, STAT1-dependent transactivation of both these promoter elements cooperates for the enhanced expression of proteins involved in anti-viral, anti-tumor, and also in pro-inflammatory mechanisms, including CCL2, CCL5, and the CXCR3 ligand CXCL10 [24, 25].

In our search for a finer definition of the mechanisms underlying the skin inflammatory condition triggered by anti-EGFR drugs, we collected evidence that these agents induce an IRF1-mediated activation of the type I IFN signalling pathway. These events could be reproduced by a MEK-selective inhibitor. Up-regulated expression of anti-viral and pro-inflammatory effectors are among their downstream consequences.

## RESULTS

### The EGFR inhibitor PD168393 perturbs TNF-α-driven gene expression and induces a type I IFN signature

In our search for pathogenic mechanisms underlying anti-EGFR drug-driven skin inflammation, we applied a whole-genome gene expression screening approach by Illumina microarrays (GSE74407), intentionally focusing on the combined use of the EGFR tyrosine kinase inhibitor PD168393 (PD16) and TNF-α rather than on the tyrosine kinase inhibitor alone. In doing so, we wanted to magnify gene expression perturbation by the use of this well-characterized experimental condition [9–12], thereby preventing possible sensitivity limits known to be associated to the microarray technique when compared to other techniques, including quantitative real-time RT-PCR [26, 27]. Normal human skin keratinocytes were treated with TNF-α for 6h, with or without co-incubation with the EGFR small-molecule inhibitor PD16. Class comparison was performed by application of the univariate two-sample *t*-test to the expression values of the 47,199 transcripts recognized by the Illumina beadchip microarrays in a low stringency analysis (*p<*0.005), or to a fraction of 9,022 transcripts selected by intensity/quality expression filtering in a high stringency analysis (*p<*0.001). By application of these two distinct analysis criteria, differentially expressed genes in the comparison between TNF-α and untreated control allowed the generation of Supplementary Table S1 with total 772 dysregulated transcripts, and Supplementary Table S2 restricted to 106 of these transcripts, respectively. Genes in Supplementary Table S2 were then filtered for Gene Ontology-annotated genes only with a >2.0 or <0.5 fold change, and the resulting 38 genes were eventually clustered in biological processes (Table 1). The most significant biological process activated by TNF-α at 6h time-point was cell-cell signaling as expected, with upregulated expression of a number of genes encoding for proteins of the IL-1 system (IL-1β, IL-36γ, IL-1ra, IL1R2), GM-CSF, the TNF-superfamily subunit lymphotoxin beta and chemokines (IL-8, CXCL10, CCL27, CCL20). Signal transducers of the TNF receptor-activated cascade (C1QTNF1, TNFAIP2, TNFAIP3, IRAK2, TNIP TRAF1), and NF-κB-responsive genes (RELB and NFKIA) were also found highly significantly up-regulated. Finally, a small group of genes encoding for proteins involved in extracellular matrix organization and disassembly, including the matrix metalloproteinases (MMP) 9 and 10 and the heparan sulphate-rich proteoglycan syndecan 4 (SDC4), were among the most strongly up-regulated genes in response to TNF-α (Table 1).

**Table 1:** Differentially expressed genes in normal human keratinocytes treated with TNF-α versus control untreated condition, clustered into biological processes

Biological Process	Up-regulated genes (fold change >2.0)	Down-regulated genes (fold change < 0.5)
Immune cell-cell signaling	IL1F9, IL8, CXCL10, IL1B, CCL27, CSF2, LTB, IL1R2, CD83, IL1RN, ECGF1, CCL20	CXCR7
TNF-mediated signaling pathway/ NFkB signaling	C1QTNF1, TNFAIP3, NFKBIA, IRAK2, TNIP1, RELB, TRAF1, TNFAIP2	
Extracell. matrix organization	LEPREL1, SDC4, MMP9, MMP10	
Regulation of endopeptidase activity	SERPINB1, SERPINB2	
Regulation of transcription/Signal transduction	PRDM1, STAT5A	ID3/VAV3
Apoptotic process	OLR1, BID	
Keratinocyte differentiation	SPRR2D, SPRR2A	
Transmembrane transport	TAP1	
Oxidation-reduction process	SOD2	
Autophagy	DRAM1	

Only transcripts of annotated genes with FDR-adjusted *p* values ≤0.001 and fold-change values ≥2.0 or ≤0.5 were considered

By contrast, statistical analysis performed with the two stringency criteria described above and aimed at the identification of differentially expressed genes in the comparison between PD16+TNF-α and TNF-α gave rise to a much more articulate picture, with 2,947 differentially expressed transcripts (*p<*0.005, Supplementary Table S3), or a selected fraction of 545 highly significant differentially expressed transcripts (*p<*0.001, Supplementary Table S4). Finally, 92 up-regulated and 104 down-regulated Gene Ontology annotated genes were clustered according to Table 2. Quite unexpectedly, the most enriched group of up-regulated transcripts belonged to the type I IFN response, including transcription factors (STAT1, STAT2) and a number of effectors of the innate antiviral response [28, 29] (Table 2). Among these, the IFN-induced proteins with tetratricopeptide repeats (IFIT) 1, 2 and 3 possess a broad-spectrum activity which includes inhibition of viral replication and cell apoptosis [30]. In addition, up-regulated expression of several enzymes involved in the cytochrome P450-mediated xenobiotic metabolism and in distinct oxidation-reduction processes, as well as some markers of cell cycle arrest and autophagy, were also peculiar of this experimental condition. In keeping with the central role of EGFR in the control of keratinocyte gene expression homeostasis [31], transcription, signal transduction, transmembrane transport, apoptotic process and small-molecule metabolic process, underwent complex perturbation, whereas other processes were dramatically suppressed, mainly including ribosome assembly and nucleologenesis, cytoskeleton organization, mitotic cell cycle and keratinocyte differentiation (Table 2). In the immune cell-cell signalling cluster, transcripts encoding for several cytokines and chemokines significantly up-regulated by TNF-α alone (Table 1), including members of IL-1 superfamily, GM-CSF, CXCL8 and CCL20 were found suppressed by PD16+TNF-α, while lymphotoxin beta and CXCL10 were further induced (Table 2). Finally, the extracellular matrix metalloproteinases MMP9 and MMP10, but not SDC4, were down-regulated upon PD16+TNF-α treatment (Table 2).

**Table 2:** Differentially expressed genes in normal human keratinocytes treated with PD16 and TNF-α versus TNF-α alone, clustered into biological processes

Biological Process	Up-regulated genes (fold change >2.0)	Down-regulated genes (fold change < 0.5)
Type 1 interferon response	IFIT2, IFIT3, IFIT1, GBP2, NAV2, IFI44L, HERC5, STAT1, IFITM1, RSAD2, ANG, OASL, IFIH1, XAF1, OAS1, STAT2, MMP7, RNASE4	
Xenobiotic metabolic process	CYP1A1, CYP1B1, ALDH3A1, CYP1A2, AKR1C3, AKR1C4, AKR1C2, UGT1A6	
Oxidation-reduction process	LOXL4, DHRS3, MOXD1, SOD2, SEPX1, NCF2, PIR	
Cell cycle arrest	IGFBP3, CDKN2B, BTG1, DST, CDKN1C	
Autophagy	GABARAPL1, ULK1, RAB9A	
Regulation of transcription	NCOA7, ATF3, MAF, ELF3, NUPR1, SOX4, BCL11B, NCOA3, HBP1, BCL6, MXD1, ARID5B, ETS2, STK16	EGR1, PPRC1, PUS1, MYC, HSPA8, PTK6
Signal transduction	VAV3, P2RY5, RGMA, MAP3K8, STARD13, SH2D3C, C1S, ADM, VSNL1, TESK2	DCBLD2, TRIB1, SPRY4, HPCAL1, ERRFI1, TRIB3, ADORA2B, SH2B3
Transmembrane transport	SLC47A2, SLC2A12, SLC7A2, TSC22D3, SLC6A9, FXYD3, KCNS1	SLC20A1, ANTXR2, LC43A3, SLC16A3, ABCC3, STEAP1
Apoptotic process	TXNIP, PIK3IP1, TNFSF10, DAPL1, ID3, TNFAIP8, ERBB3	PHLDA2, PHLDA1
Small mol. metabolic process	KMO, KYNU, GCLC, STARD5, CROT	CYP27B1, MAT2A, UCK2, UPP1, HK2, MTHFD2, XDH, PLA2G3, PDSS1
Immune cell-cell signalling	LTB, CXCL10, CCL2, CXCR7	HBEGF, IL1R2, IL1A, CD276, F3, CSF2, TGFA, IL8, IL4R, IL24, NRG1, IL1F9, TGFBR2, IL1RN
Ubiquitin-assoc. proteolysis	FBXO32, EFNA1	XBP1
Protein dephosphorylation	DUSP1	DUSP6, DUSP5, PTPN12
Extracell. matrix organization	LUM	HAS3, HS3ST2, ITGA5, THBS2
Protein translation		AIMP2, EIF2B2
Peptidase activity		MMP9, STAMBPL1, MMP10, SERPINB1
Keratinocyte differentiation		JAG1, ETV5, SPRR2A, SPRR2F, SPRR2D, TGM2, FABP5
Cell proliferation, Mitotic cell cycle		LYAR, NP, CCND1, CTPS, BYSL, CDC25A, PPAT, CDK5R1, CCND2
Cytoskeleton organization		PLEK2, GJB2, FLNB, GJB6, ARHGAP25, FERMT1, FSCN1, PAK6, TMEM158, OSBP2, CALM1, PLEKHG3, RAI14
Ribosome assembly/biogenesis and nucleologenesis		DDX21, RRS1, HSPC111, GNL3, DKC1, NOLC1, RRP12, NIP7, METTL1, URB2, NOP2, GTPBP4, BOP1, RPF2, PNO1, BRIX1

Only transcripts of annotated genes with FDR-adjusted *p* values ≤0.001 and fold-change values ≥2.0 or ≤.5 were considered

### Quantitative real-time RT-PCR assays confirm microarray data

**Figure 1:**
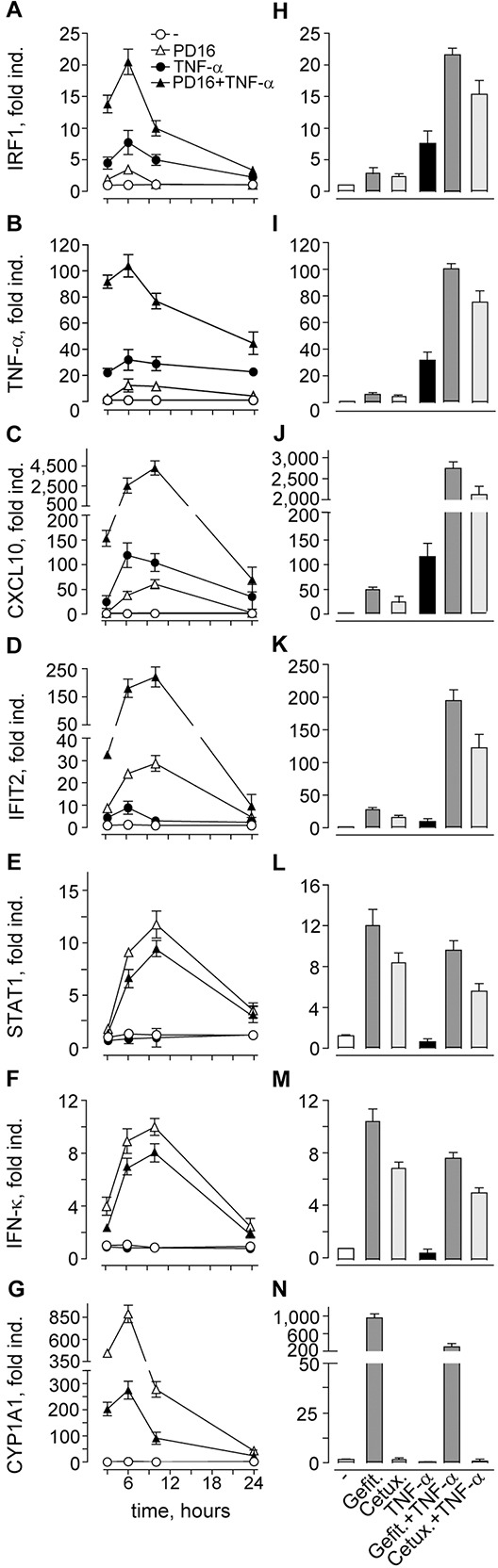
Quantitative real-time RT-PCR assays confirm microarray data **A-G**. Cells were treated with 2 μM PD168393 (PD16), with or without immediate addition of 50 ng/ml TNF-α, for the indicated intervals. **H-N.** Transcript measurements at 6h time-point. Cells were treated with gefitinib (Gefit. 2 μM) or cetuximab (Cetux., 25 μg/ml), with or without immediate addition of TNF-α. Data are expressed as the mean ± S.D. (*n* = 4 *per* condition) of transcript fold induction (fold ind.). Data are representative of three independent experiments.

SYBR Green-based quantitative real-time RT-PCR was performed to verify our microarray data, and analyze time-dependent changes in the expression of a small cluster of differentially expressed transcripts. This cluster comprised genes highly differentially expressed in response to PD16+TNF-α *vs* TNF-α, including the most strongly (i.e. 26.7-fold) up-regulated transcript of the cytochrome P450 superfamily member CYP1A1, the chemokine CXCL10, and the type I IFN response genes IFIT2 and STAT1 (*p<*0.001, Table 2). We also included the transcripts for the cytokine TNF-α, the transcription factor IRF1, and the keratinocyte-specific type I IFN-κ, all significantly upregulated (>2-fold change) according to low stringency analysis (*p<*0.005, Supplementary Table S3). Noteworthy, our microarray data clearly indicated that IFN-κ was the only type I IFN that could be found constitutively expressed in our Illumina beadchip data in unstimulated keratinocytes (mean IFN-κ transcript expression values in triplicate unstimulated controls: 299.77; background noise: <150), while all IFN-α isoforms and IFN-β1 were undetectable (GSE74407). Quantitative real-time RT-PCR showed that TNF-α induced the transcription of IRF1, TNF-α, CXCL10 and IFIT2, with peak values at 6h (Figure 1A–1D). These genes were induced also by PD16 alone, but their expression was dramatically enhanced by concomitant administration of PD16 and TNF-α, with a shift in the peak of CXCL10 and IFIT2 to 10h (Figure 1C and 1D). By contrast, PD16 only could induce STAT1, IFN-κ and CYP1A1 gene expression, which was significantly reduced by TNF-α co-administration (Figure 1E–1G). Finally, these molecular events could be reproduced by the small-molecule EGFR inhibitors gefitinib (2 μM) and erlotinib (2 μM, not shown), and by the anti-EGFR antibody cetuximab (25 μg/ml) (Figure 1H–1N), with this last drug acting extracellularly and hence ineffective on CYP1A1 induction (Figure 1N). In their whole, these data confirmed that EGFR inhibition *per se* triggers the expression of pro-inflammatory genes, and generates a type I IFN response in normal human keratinocytes. The pro-inflammatory cytokine TNF-α enhanced distinct components of this response, as observed for IRF1, TNF-α, IFIT2 and CXCL10, but opposed others, including STAT1 and IFN-κ.

### IRF1 and STAT1 are activated in response to EGFR or MEK inhibition

**Figure 2:**
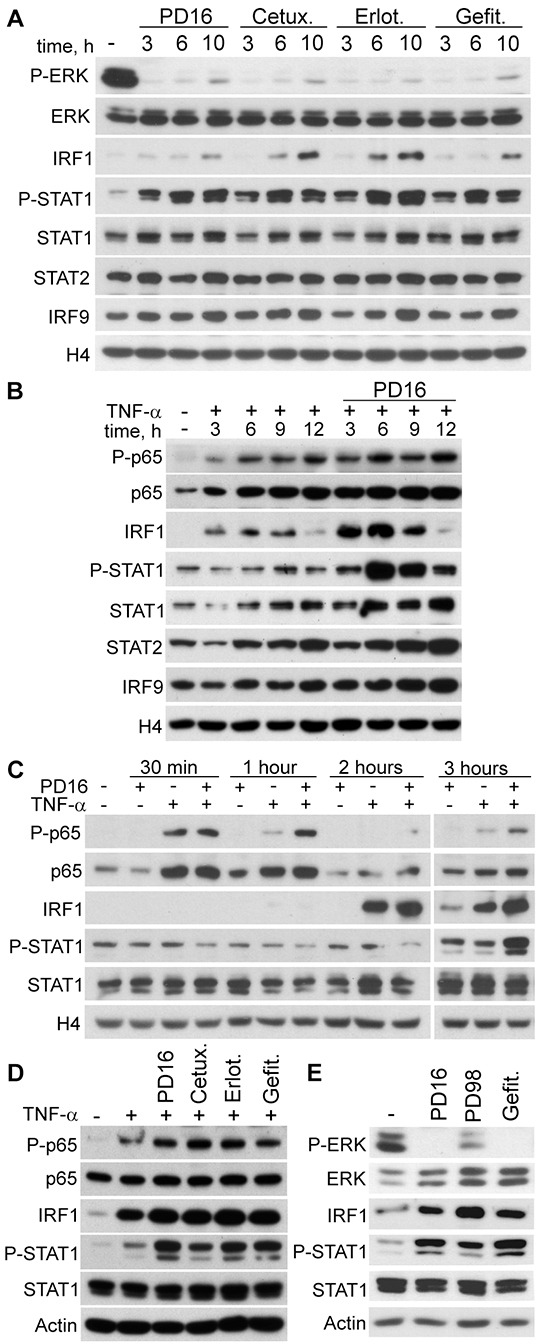
IRF1 and STAT1 are activated in response to EGFR or MEK inhibition Western blot analysis was performed in nuclear protein subfraction **A-C.** and in total cell lysate **D, E.** of human keratinocytes. A. Cells were treated with PD168393 (PD16), erlotinib (Erlot.), gefitinib (Gefit.), all at 2 μM, or cetuximab (Cetux., 25 μg/ml) for the indicated time intervals. B, C. TNF-α (50 ng/ml), with or without immediate addition of PD16, was administered for the indicated time-points. D. Cells were treated with the EGFR inhibitors and/or TNF-α for 6h. E. Cells were treated with the EGFR inhibitors or the MEK inhibitor PD98059 (PD98, 20 μM) for 6h. P-ERK, Phospho-Thr202/Tyr204-ERK1 and Phospho-Thr185/Tyr187-ERK2; P-STAT1, Phospho-Tyr701-STAT1. P-p65, Phospho-Ser536-p65. Histone 4 (H4) and actin were used for loading control of nuclear subfraction or total cell lysates, respectively.

Inhibition of EGFR signalling implies profound inactivation of the Ras/Raf/MEK/ERK pathway in epithelial cells, including skin keratinocytes [32]. A considerable body of independent observations indicates that this pathway exerts a negative regulation on type I IFN response [33, 34], with IRF1 recognized among its molecular targets [35–37]. Anti-EGFR drugs induced a strong suppression of ERK phosphorylation, as expected (Figure 2A). IRF1 was barely detectable in the nuclei of unstimulated keratinocytes, and underwent a slow accumulation in response to the anti-EGFR treatments, while high levels of phosphorylated STAT1 (P-STAT1) could be observed throughout the 3-10h interval (Figure 2A). Both STAT2 and IRF9 were present in unstimulated cells. Similar to STAT1, IRF9 levels tended to increase with the treatments. Also the NFκB subunit p65 was abundant in the nuclear lysates, but no P-p65 could be detected following anti-EGFR drugs (data not shown). By contrast, keratinocytes responded to TNF-α with a long-lasting increase of nuclear P-p65, while IRF1 was detected in the 3-9h interval and P-STAT1, STAT1, STAT2 and IRF9 displayed a rather modest increase (Figure 2B). Importantly, combination of PD16 and TNF-α led to a strong enrichment of nuclear IRF1 and P-STAT1, both peaking at 6h, and also of STAT1, STAT2 and IRF9 (Figure 2B). Some enhancement of P-p65 at 6 and 12h could also be reproducibly observed (Figure 2B). An analysis of these molecular events at earlier time-points showed TNF-α-driven P-p65 nuclear translocation peaking at 30 min and almost vanishing at 1h, whereas this signal persisted with PDl6+TNF-α (Figure 2C). Analogously, TNF-α-induced nuclear IRF1 peaked at 2h but almost disappeared at 3h, while it was still high following PD16+TNF-α at this time-point. Increase of P-STAT1 was detected only at 3h stimulation following TNF-α, but underwent a strong improvement with PD16+TNF-α (Figure 2C). Enhanced P-p65, IRF1 and P-STAT1 levels were still detectable after 6h treatment with anti-EGFR agents combined to TNF-α, as observed in total cell lysates (Figure 2D). Finally, up-regulated IRF1 and P-STAT1 were also induced by the selective MEK inhibitor PD98059 (Figure 2E). These results documented that human keratinocytes respond to anti-EGFR drugs or MEK inhibition with a delayed, sustained activation of transcription factors characteristic of type I IFN signalling pathways. In addition, a robust synergism is evoked when anti-EGFR drugs are associated to TNF-α.

### IRF1 and STAT1 are functionally involved in PD16-driven gene expression

**Figure 3:**
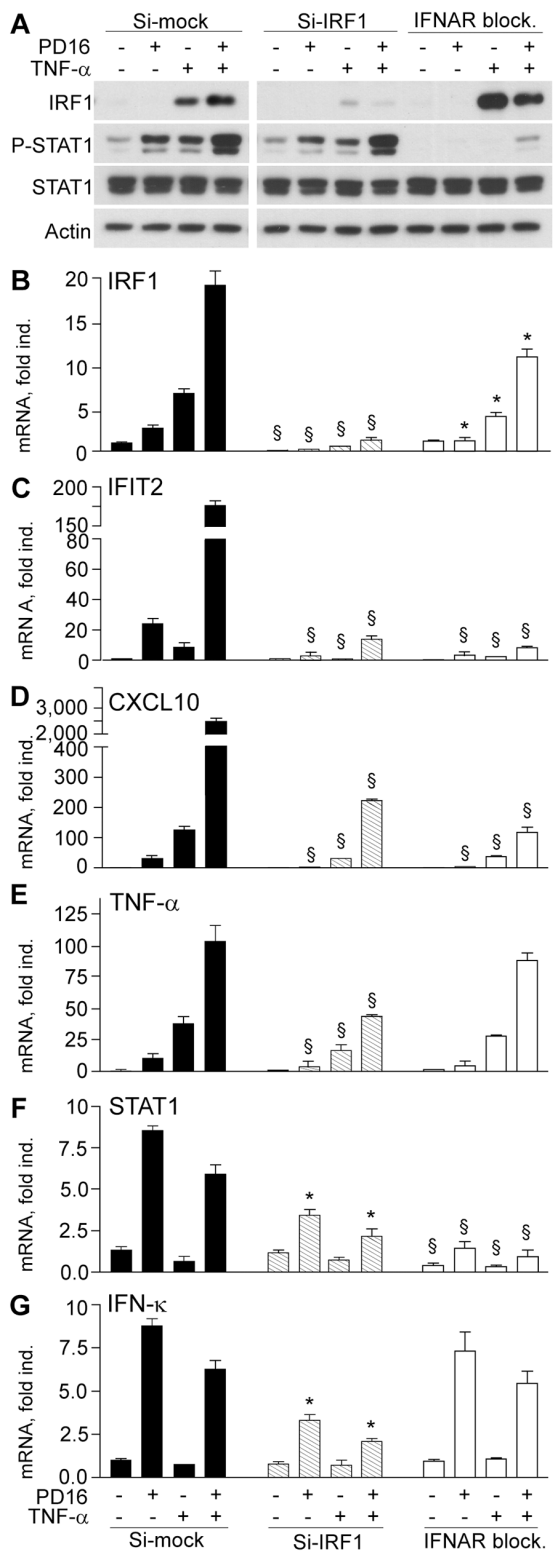
IRF1 transcript silencing and IFNAR blockade impair anti-EGFR-driven gene expression **A.** Western blot analysis of total cell lysates from keratinocytes stimulated with PD16 (2 μM), TNF-α (50 ng/ml), or their combination for 6h. Keratinocytes received these treatments two days after transfection with irrelevant small interference RNA (Si-mock), with IRF1-targeted siRNA (Si-IRF1), or soon after addition of 5 μg/ml anti-IFNAR2 blocking antibody (IFNAR block.). **B-G.** Quantitative real-time RT-PCR assessment of transcript levels. Data are expressed as the mean ± S.D. (*n* = 4 per condition) of transcript fold induction (fold ind.). *, *p*<0.05, and ^§^, *p*<0.01 *versus* same treatment (untreated controls, PD16, TNF-α, or PD16+TNF-α) in the Si-mock cultures.

In order to functionally confirm IRF1 and STAT1 direct involvement in keratinocyte gene expression response upon EGFR blockade, we performed experiments of selective impairment of these two transcription factors. IRF1-targeted specific small interference RNA (Si-IRF1) impaired both IRF1 transcription and translation (Figure 3A and 3B), but also led to strong suppression of IFIT2, CXCL10 and TNF-α transcription in response to PD16, TNF-α or their combination (Figure 3C–3E). In addition, a significant reduction of PD16-driven STAT1 and IFN-κ gene expression was observed (Figure 3F–3G), indicating that IRF1 is critically implicated in the transcription of all these genes. Effective abrogation of both constitutive and induced STAT1 phosphorylation (Figure 3A), but also suppression of both basal and induced STAT1 gene transcription (Figure 3F) were observed in response to anti-IFNAR2 blocking antibody (IFNAR block.), indicating that functional IFNAR is required for both these events. Despite whole-cell protein levels of IRF1 (Figure 3A), we reproducibly observed a significant decrease in PD16- and/or TNF-α-induced expression of this transcription factor in the absence of functional IFNAR (Figure 3B). This experimental condition also associated to a profound suppression of IFIT2 and CXCL10 (Figure 3C and 3D) and a tendency to modestly reduce TNF-α gene expression (Figure 3E), while it did not perturb IFN-κ transcription (Figure 3G).

### Anti-EGFR drugs enhance the expression of IFN-κ

**Figure 4:**
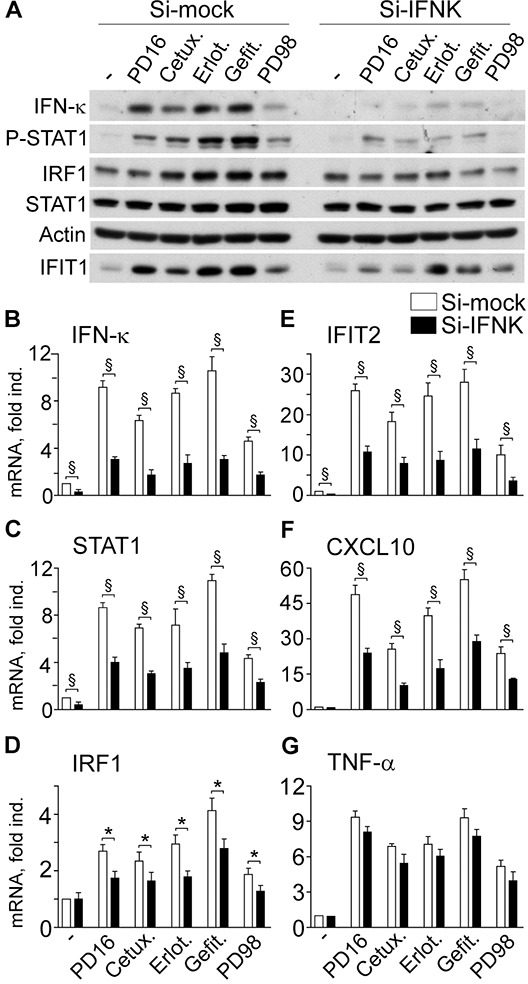
IFN-κ transcript silencing (Si-IFNK) affects anti-EGFR- or anti-MEK-driven gene expression dysregulation **A.** Western blot analysis of total protein lysates from cells stimulated with PD168393 (PD16, 2 μM), Cetuximab (Cetux., 25 μg/ml) Erlotinib (Erlot., 2 μM), Gefitinib (Gefit. 2 μM) or PD98059 (PD98, 20 μM) for 6h. Keratinocytes received these treatments two days after transfection with irrelevant small interference RNA (Si-mock) or INF-κ-targeted siRNA (si-IFNK). **B-G.** Quantitative real-time RT-PCR assessment of transcript levels. Data are expressed as the mean ± S.D. (*n* = 4 per condition) of transcript fold induction (fold ind.). *, *p*<0.05, and ^§^, p<0.01.

In keeping with our data of enhanced IFN-κ gene transcription, we observed strong up-regulation of IFN-κ protein expression in response to anti-EGFR drugs and, although more modestly, to the experimental anti-MEK agent PD98059 (Si-mock, Figure 4A). Anti-IFN-κ transcript-specific small interference RNA (Si-IFNK) impaired IFN-κ transcription (Figure 4B) and translation, along with deep suppression of P-STAT1 and reduction of induced IRF1 (Figure 4A). Also anti-EGFR drug- or anti-MEK-induced IFIT1 protein was decreased in IFN-κ-silenced keratinocytes (Figure 4A). Down-regulation of IRF1 and, more significantly, of STAT1, IFIT2 and CXCL10, and a weak reduction of TNF-α transcription could be detected (Figure 4C–4G), directly confirming the involvement of IFN-κ in human keratinocyte response to EGFR or MEK inhibition.

### IFN-κ is up-regulated in the lesional skin of patients treated with cetuximab

**Figure 5:**
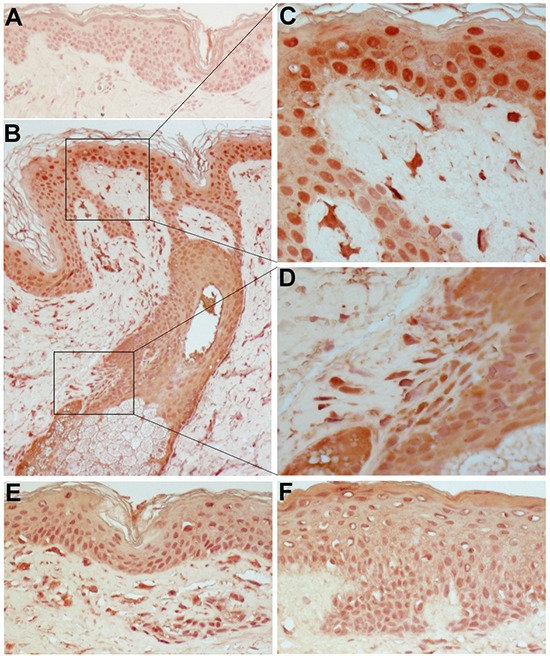
IFN-κ is highly expressed in the lesional skin of patients treated with cetuximab Immunohistochemical staining was performed on normal skin **A.** and on skin lesions from three distinct patients under treatment with Erbitux® (cetuximab) **B, E, F.** Original magnification: x200. **C.** and **D.** are higher magnification photographs (x400) of particulars of B. Representative results of five independent staining experiments.

We found IFN-κ highly expressed in the lesional skin of patients treated with cetuximab (Figure 5B, 5E, and 5F). In particular, compared to the weak, diffuse staining in the epidermis of healthy skin (Figure 5A), a strong IFN-κ-specific positivity was detected in most keratinocytes throughout the whole epidermis and also in dermal cell populations, including dendritic cell-like bodies infiltrating the upper dermis (Figure 5B, 5C and 5E). Intense IFN-κ staining was also associated to the damaged layers of keratinocytes at the initial stage of a pilosebaceous unit destruction (Figure 5B and 5D). Positivity was retained even in the cetuximab-induced spongiotic epidermis (Figure 5F), a histopathologic feature of acute dermatitis characterized by scattered intraepidermal fluid-containing spaces due to keratinocyte apoptosis [38].

## DISCUSSION

Here we presented evidence that anti-EGFR drugs autonomously trigger activation of signal transduction pathways responsible of enhanced expression of IFN-κ, leading to persistent activation of STAT1 and consequent induction of the antiviral native immunity and of the inflammatory response in human keratinocytes. In the skin lesions of patients under treatment with cetuximab, strong expression of IFN-κ characterized all layers of epidermal keratinocytes and also leukocyte populations infiltrating the upper dermis. This observation indicates that by a direct action of cetuximab, and/or by keratinocyte-derived cytokine/chemokine milieu, type I IFN response conditions the whole immune response in these lesions.

High stringency class comparison analysis of our Illumina microarrays highlighted a rather limited number of highly significantly perturbed genes in response to TNF-α in normal human keratinocytes. By contrast, co-administration of PD16 and TNF-α led to an articulate alteration of TNF-α-driven keratinocyte transcriptional program, in keeping with the central role of EGFR signalling in epithelial cell biology [31]. Up-regulated expression of a small cluster of cytokines (including TNF-α and beta subunit of lymphotoxin) and chemokines (including CCL2 and CXCL10), but suppression of distinct TNF-α-induced mediators (including CXCL8 and GM-CSF), confirmed previous findings from keratinocyte cultures *in vitro*, animal models *in vivo*, and skin lesions of patients under treatment with anti-EGFR drugs *ex vivo* [8, 10–12, 14]. Quite unexpectedly, class comparison analysis clearly indicated that the combination of TNF-α and EGFR inhibition induced upregulated expression of a relevant number of genes characteristic of type I IFN signalling pathway, including the transcription factors STAT1 and 2, and a cluster of effectors involved in viral clearance. Among these antiviral effectors, IFIH1/MDA5 is a pattern recognition receptor involved in the detection of cytosolic viral double-stranded RNA and RNA intermediates; IFITM1 inhibits viral entry by contrasting the fusion of the viral and cellular membranes; IFIT1, 2, and 3 mainly act by inhibiting distinct steps of the translation of viral mRNA; also OAS1, OASL, GBP2, RNASE4 and HERC5 are involved in the inhibition of RNA virus translation and replication, while RSAD2/viperin is active in the early as well as in the late (egress) stages of virus life cycle [18, 29]. Beyond their antiviral functions, independent observations suggest that some of these proteins may mediate tissue damage during cell response to type I IFNs. In particular, IFITs possess anti-proliferative and pro-apoptotic activities [30, 39]. Experiments designed to validate our microarray data both at the transcript and protein level confirmed that type I IFN signalling activation was not restricted to the combination of TNF-α and PD16, since it could be reproduced by anti-EGFR drugs alone.

Mechanistically, we observed that anti-EGFR drugs induced *de novo* gene expression and progressive nuclear enrichment of IRF1, and robustly synergized in this process with TNF-α, in its turn an inducer of IRF1. Silencing experiments confirmed IRF1 involvement in the expression of CXCL10, TNF-α and IFIT2 upon PD16 and/or TNF-α stimulation. They also showed that IRF1 expression was implicated in the induction of IFN-κ, with TNF-α totally ineffective and even opposing anti-EGFR drug-induced expression of this cytokine. In the literature, TNF-α-driven IRF1 activation was shown to upregulate IFN-β, leading to sustained STAT1-dependent expression of antiviral effectors and pro-inflammatory mediators, including TNF-α, CCL2 and CXCL10 in primary macrophages [40] and endothelial cells [41]. In contrast with these reports, our data suggest that MEK/ERK suppression is required for IRF1 and/or its partner(s) to transactivate IFN-κ gene in normal human keratinocytes. A considerable body of independent observations actually confirms that MEK/ERK activity exerts a negative regulation on IRF1 transcriptional activity and eventually on type I IFN response [33, 34], and that this regulation can be effectively reverted by MEK/ERK inhibition [35–37]. All these reports are based on cells with a strong MEK/ERK activation due to oncogenic Ras [33, 36–37] or to viral infections [34, 35]. EGFR-dependent down-regulation of IRF1 and, as a consequence, of IRF1-dependent type III IFN-λ, was shown to be the strategy exploited by some respiratory viruses to suppress the antiviral response in human airway epithelial cells, a mechanism effectively reverted by EGFR inhibition [42]. Finally, very similar to what we found in keratinocytes, cetuximab induced type I IFN/STAT1 signature in A431 human epidermal carcinoma cell line harbouring oncogenic Ras, with enhanced transcription of STAT1, a number of antiviral effectors (IFIT1, IFITM1, RSAD2, OAS1, and OAS2 among others), and also pro-inflammatory mediators, including TNF-α and very high lymphotoxin beta [43]. From these and our own findings, we can reasonably hypothesize that sustained activity of the EGFR/Ras/Raf/MEK/ERK phosphorylation cascade might exert a permanent, tonic regulation on IRF1 functional state, leading to effective control on the transactivation of a subset of genes which includes IFN-κ. To our knowledge, the mechanism of this regulation has not been clarified yet.

Type I IFN signalling is constitutively active in many cell types, including epithelial cells. Previous reports [20, 44] and our own evidence indicate that unstimulated keratinocytes express low levels of IFN-κ. Through this pathway, cells maintain basal expression of STAT1 and its downstream targets, in their turn involved not only in the antiviral defence and in the immuno-activation, but also in cell survival, cell cycle regulation and differentiation [17]. Impairment of the EGFR/Ras/Raf/MEK/ERK cascade by anti-EGFR, anti-Raf, or anti-MEK anti-cancer drugs could enhance type I IFN response, giving rise to inflammatory events in the skin and possibly also in the intestinal tract, the two most important targets of their adverse events [13]. Indeed, type I IFNs frequently do cause dermatological side effects [45]. Subcutaneous injections of IFN-β in multiple sclerosis patients were shown to initiate an inflammatory skin reaction characterized by enhanced CCL2, CCL5 and CXCL10 in keratinocytes and infiltrating leucocytes [15], actually the same chemokines strongly up-regulated in response to EGFR inhibition in human and mouse keratinocytes, *in vitro* and *in vivo* [8–11, 14]. Analogously, *in vivo* evidence suggests that type I IFNs may have a pro-inflammatory activity also in the colonic epithelium [46], including IFNAR-mediated enhanced expression of TNF-α, CCL2 and CXCL10 and consequent massive infiltration of leukocyte populations involved in tissue damage [47, 48]. Nonetheless, in the context of epithelial malignancies, these mechanisms could provide a further anti-cancer activity concomitant to apoptosis promotion, due to improved recruitment of functional antigen presenting cells and cytotoxic T cells within neoplastic lesions [39, 49].

We also observed that abrogation of IFN-κ functional interaction with its receptor by IFNAR2-targeted neutralizing antibody, or IFN-κ silencing, similarly suppressed both constitutive and induced STAT1 gene expression and phosphorylation, with consequent strong impairment of IFIT2 and CXCL10 transcription and a rather milder, although statistically significant, reduction of IRF1, but no relevant perturbation of TNF-α. Notably, despite its inability to enhance IFN-κ gene expression, we reproducibly observed TNF-α-induced STAT1 phosphorylation and nuclear translocation. These events were suppressed by IFNAR2 blockade, indicating that they were fully dependent on the formation of the type I IFN/IFNAR signalling complex. Among all type I IFNs, only IFN-κ and IFN-β are heparin binding cytokines [20]. When released in the extracellular milieu, they establish a high affinity interaction with the extracellular heparan sulphate chains of membrane proteoglycans, mainly including SDCs [50], and hence they are practically undetectable in cell supernatants [51]. This binding keeps IFNs close to the site of secretion, but in the same time finely tunes their interaction with IFNAR [52]. Our microarray data showed that SDC4 gene expression was highly significantly up-regulated in response to TNF-α with or without PD16. Hence, it is possible that TNF-α triggers shedding of pre-formed SDC4-bound IFN-κ and eventual IFNAR transactivation *via* a triple-membrane-passing signal [53], in the absence of *de novo* IFN-κ expression. TNF-α was already shown to exploit such a mechanism in human keratinocytes, with MMP-mediated shedding of the mature forms of the EGFR ligands and consequent EGFR transactivation [10]. Further investigation is however required to confirm the existence of a TNF receptor-IFNAR cross-talk.

Our data show that anti-EGFR drugs induce IFN-κ in normal human keratinocytes and this cytokine is implicated in the enhanced expression of pro-inflammatory mediators. Strong IFN-κ immunoreactivity in the lesional skin of patients undergoing anti-cancer therapy with cetuximab suggests that these mechanisms are active *in vivo*. In these lesions, IFN-κ was not confined to keratinocytes but also stained the inflammatory infiltrate of the upper dermis including dendritic-shaped cells, indicating that anti-EGFR-dictated activation of type I IFN response involves leukocyte populations. This could be due to a direct effect of the drug on these cells, or, indirectly, to keratinocyte-generated local cytokine/chemokine milieu, active both in their recruitment and activation towards a type I IFN response. Enhanced IFN-κ expression in the epidermis and in dermal monocytes and dendritic cells was previously described in lesions of patients with chronic inflammatory skin disorders [20, 44]. *In vitro*, IFN-κ or IFN-β was shown to enhance dendritic cell release of TNF-α and IL-10 [20]. Of relevance, IL-10 is a well-recognized regulatory cytokine, with suppressive effects on dendritic cell functional maturation [54] and on keratinocyte expression of the anti-microbial peptides beta-defensin-2 and -3 and cathelicidin LL37 [55, 56], whose synergistic activity leads to effective killing of *Staphylococcus aureus* [57]. Hence, abnormal activation of type I IFN response in the infiltrate could misdirect the immune response and further aggravate the defective expression of antimicrobial peptides due to EGFR inhibition in keratinocytes [8], leading to high susceptibility to *Staph. aureus* skin infections in patients undergoing treatment with these EGFR inhibitors [8, 58]. Mounting evidence actually indicates that type I IFNs can exert a deleterious effect in bacterial infections [16, 59], and may be involved in harmful rather than beneficial functions [60]. For instance, type I IFNs are considered crucially implicated in the increased susceptibility to post-influenza *Staph. aureus* respiratory superinfections [61, 62], a tremendous cause of human morbidity and mortality.

Many viruses activate EGFR and exploit EGFR-mediated signalling for critical steps in their life cycle, including cell entry, replication, and viral antagonism to the host anti-viral systems, and EGFR inhibition has been proposed as a novel approach for prevention and/or treatment of viral infection [63]. Notably, despite their strong suppressive effect of antibacterial innate immunity, many reports document that anti-EGFR drugs do possess anti-viral activity. Erlotinib impairs infection by all major hepatitis C virus genotypes and viral escape variants in cell cultures and in a human liver chimeric mouse model *in vivo* [64, 65]. Erlotinib also diminishes infectivity of specific strains of syncytial virus in respiratory epithelial cell cultures and in mouse models in vivo [66]. Analogously, gefitinib was shown to suppress influenza A virus and rhinovirus infection of bronchial epithelial cells and in mouse models [42]. These respiratory viruses suppress the antiviral defence of airway epithelium by inducing EGFR activation, leading to IRF1 suppression and consequent loss of type III IFN-λ, a mechanism that is reverted by EGFR inhibition [42]. Human keratinocytes infected with high-risk papillomaviruses were shown to possess a reduced capacity to attract immune cells due to EGFR-dependent impairment of chemokine expression, including CCL2 and the CXCL10 homologue CXCL9, an event that was restored by cetuximab [67]. Since papillomaviruses were shown to repress IFN-κ to prevent pathogen recognition receptor and antiviral-gene expression [22], the possibility exists that cetuximab might exert its pro-inflammatory and antiviral activity essentially by enhancing IFN-κ expression in infected keratinocytes.

Our data emphasize the regulatory role of the EGFR/Ras/Raf/MEK/ERK on type I IFN response in normal human keratinocytes. Aberrant activation of this response by impairment of the EGFR-driven pathway may lead to the establishment of a pro-inflammatory milieu in the skin and presumably in other epithelia, eventually precipitating unwanted tissue damage and susceptibility to bacterial infections. Since these mechanisms are likely to be instrumental to the anti-cancer efficacy of EGFR-, Raf- and MEK-directed inhibitors, improvement in the targeted delivery of these drugs to malignant cells represents a valid strategy in the effort to limit their *off target* toxicity [68].

## MATERIALS AND METHODS

### Ethics statement

These studies were conducted according to the Declaration of Helsinki Guidelines and were approved by the institutional review boards of the Istituto Dermopatico dell'Immacolata (healthy donors and patients affected by psoriasis) and the University of Chieti and Pescara, Italy (patients treated with anti EGFR therapy), as previously specified [12]. Informed consent was obtained from all participants included in the study.

### Patients and healthy donors

Paraffin-embedded biopsy specimens from skin lesions of patients under treatment with cetuximab, psoriasis lesions and normal human skin were previously described [12]. Briefly, four-mm punch biopsies were taken from lesional skin of adult patients with a mild to moderate cetuximab-associated papulo-pustular skin rash (n = 8, three females and five males, age 50-66), chronic plaque psoriasis (n = 5, two females and three males, age 30-48) and from normal skin of healthy subjects (n = 7, three females and four males, age 24-59). Epidermal sheets for keratinocyte cultures were obtained from healthy individuals undergoing plastic surgery (mammoplasty or abdominoplasty) (n = 4, two females and two males, age 25-40).

### Keratinocyte cultures and treatments

Primary cultures of normal human keratinocytes were obtained as previously described [69], and routinely grown in serum-free Keratinocyte Growth Medium from Lonza (Walkersville, MD, USA). This medium is formed of an essential Keratinocyte Basal Medium nutrient solution supplemented with 0.2 ng/ml EGF, 0.18 μg/ml hydrocortisone, 5 μg/ml bovine insulin, 0.2% bovine pituitary extract, and gentamicin sulfate solution. In the 24 hours preceding and during the experiments, subconfluent cell cultures were switched to Keratinocyte Basal Medium. All assays were performed on human keratinocytes obtained from at least three distinct healthy donors.

### Chemicals and reagents

The small-molecule, cell permeant EGFR kinase inhibitor PD168393 and the MEK1/2 inhibitor PD98059 were purchased from Selleckchem (Munich, Germany). Gefitinib and Erlotinib were from Cayman (La Jolla, CA, USA). All these chemicals were dissolved in dimethylsulfoxide (DMSO). In all experiments, the DMSO concentration as vehicle control was 0.1% (v/v). Cetuximab (5 mg/ml, Erbitux®) was provided by the Hospital Pharmacy at the Istituto Dermopatico dell'Immacolata. TNF-α was from R&D Systems (Milan, Italy). Where specified, we employed a neutralizing antibody targeting the human IFN-α/β receptor chain 2 (IFNAR2, CD118) (MMHAR-2, Cat. No. 21385-1, PBL Biomedical Laboratories, Piscataway, NJ).

### RNA extraction

Total RNA was extracted using TRizol reagent (Life Technologies) according to the manufacturer's instructions.

### mRNA profiling

Gene expression profiles were measured using Illumina platform on Illumina HumanHT-12 v4 Expression BeadChip (Illumina, San Diego, CA) comprising 47,199 specific probes for genes annotated on National Center for Biotechnology Information Reference Sequence Database, RefSeq Release 38 (November 7, 2009). Chip scanning was performed on an iScan system (iScan Control Software). Probe intensity signals were processed with a Genome Studio Expression Module, which allowed a per-quantile normalization. Normal human keratinocytes were treated for 6h with 50 ng/ml TNF-α, with or without concomitant administration of the experimental EGFR inhibitor PD168393 (2 μM). cRNA was obtained from total RNA, and analyzed according to the manufacturer's instructions. The complete dataset of our study is available from the National Center for Biotechnology Information Gene Expression Omnibus database (GSE74407). Gene expression profiles were analyzed using the class comparison between-groups function of BRB-ArrayTools (Richard Simon and BRB-ArrayTools Development Team) at the BioInformatics Service (Unimed Scientifica, Rome, Italy). False discovery rate was computed per gene using the Benjamini and Hochberg method [70]. The class comparison function also performs random permutations of the class labels, recomputing the *t* tests of each gene for each random permutation.

Differentially modulated genes were identified using the two- sample *t* test, with *p<*0.001 and false discovery rate <0.1 as significance thresholds. Since our arrays have a small number of samples (*n* = 3) per class, differentially expressed genes were also evaluated using the random variance version of the two-sample *t* test, as suggested by the BRB-ArrayTools user manual. In this case, differentially modulated genes were identified using the random variance version of the two- sample *t* test, with *p<*0.005 and false discovery rate <0.1 as significance thresholds, for a lower stringency analysis. Tools and databases provided by the Gene Ontology Consortium were used for functional categorization of genes into major biological processes [71, 72].

### Quantitative real-time RT-PCR

For cDNA synthesis, 1 μg of total RNA was reverse transcribed using SuperScript II Reverse Transcriptase (Invitrogen). PCR were performed in a volume of 25 μl using SYBR Green PCR Master Mix (Applied Biosystem, Foster City, CA) and 1:25-1:40 dilution of cDNA. The primer sets were designed using Universal Probe Library Assay Design Center (Roche Applied Science, Penzberg, Germany), were synthesized by Sigma Aldrich (Milan, Italy), and are listed in Supplementary Table S5. PCR products were measured by the ABI PRISM 5700 detection system (Perkin-Elmer, Norwalk, CT). Quantification was performed by the comparative CT method [73]. All determinations were performed in triplicate.

### Transfection with specific small interference (si) RNA

A pool of four SiRNA targeted against IRF1 (L-011704-00-0005), IFN-κ (L-013217-00-0005) or an irrelevant sequence (L-011511-00-0005) was purchased from Dharmacon RNA Technology (Lafayette, CO, USA). Keratinocytes cultured in 6-well plates were incubated with a mixture of 50 nM SiRNA and 4 μl/ml INTERFERin® transfection reagent (Polyplus Transfection, purchased from Euroclone, Milan, Italy), according to the manufacturer's instructions. After 48 h incubation, SiRNA-transfected keratinocytes were exposed for 6h to distinct treatments. The supernatants were subsequently collected and the cell pellet used for RNA or total protein extraction.

### Keratinocyte lysis

Total cell lysis was performed with a RIPA buffer composed of 20 mM Tris-HCl, pH 7.5, 150 mM NaCl, 1% Triton X-100, 1 mM EDTA, 1 mM sodium orthovanadate, in the presence of an antiprotease cocktail (Roche Diagnostics, Mannheim, Germany). Nuclear lysates were performed as described [74] with slight modifications. Briefly, keratinocytes were lysed in an hypotonic buffer (10 mM HEPES, 10 mM KCl, 0.1 mM EDTA, and 0.1 mM EGTA) supplemented with 1 mM sodium orthovanadate and an anti-protease cocktail. The lysate was centrifuged at 1,800xg for 10 min to precipitate nuclei. The nuclear pellet was finally lysed by an hypertonic buffer C (20 mM HEPES, 0.4 M NaCl, 1 mM EDTA, 1 mM EGTA, in the presence of 1 mM sodium orthovanadate and an antiprotease cocktail). During all the phases, cell lysates were kept on an ice bath.

### Western blot analysis

Proteins were resolved on a 10% SDS–PAGE, transferred to polyvinylidene difluoride filters (Immobilon-P; Millipore), and probed with primary antibodies, including Phospho-Tyr701-STAT1 (# 7649), Phospho-Ser536-p65 (# 3031), IRF1 (# 8478), IFIT1 (# 12082), P-ERK1/2 (# 9101), ERK1/2 (# 9102) all from Cell Signalling Technology (Beverly, MA, USA); STAT1 (# sc-346), STAT2 (# sc-476), IRF9 (# sc-10793), from Santa Cruz Biotechnology (Inc., CA, USA); IFN-κ (# H00056832-M01, clone 1B7, Abnova GmbH, Heidelberg, Germany). Anti-actin and anti-histone 4 (H4) antibodies (both from Santa Cruz) were used for loading control of total cell and nuclear cell lysates, respectively.

### Immunohistochemistry

Paraffin-embedded sections were incubated with 20E07 mAb (5 μg/ml) raised against human IFN-κ, as previously described [44]. Secondary biotinylated mAbs and staining kits (Vector Laboratories, Burlingame, CA, USA) were used to develop immunoreactivities, and 9-ethyl-3-aminocarbazole was used as substrate. Sections were finally counterstained with hematoxylin.

### Statistical analysis

The Wilcoxon signed-rank test (GraphPad prism Software, La Jolla, CA, USA) was applied to compare differences between groups of data. Significance was assumed at a *p*-value of 0.05 or less.

## SUPPLEMENTARY TABLES










