# Supplementary material for: Epidermal growth factor receptor inhibitors trigger a type I interferon response in human skin
Source: Oncotarget. 2016 Jun 14;7(30):47777–93. doi: 10.18632/oncotarget.10013 (PMC5216978; doi:10.18632/oncotarget.10013)
Supplement: Supplementary file 1 [file oncotarget-07-47777-s001.pdf]

Epidermal growth factor receptor inhibitors trigger a type I interferon response in human skin

SUPPLEMENTARY TABLES

Supplementary Table S1: Differentially expressed genes among Class 1 (treatment with TNF-a, 6 hours) and Class 2 (parallel untreated control) sorted by p-value of the univariate test. Type of univariate test used: two-sample T-test with random variance model. Number of genes for random variance estimation: 47,199. Number of genes that passed filtering criteria: 47,199. Random variance model parameters: a = 1.53912, b = 75.12785, Kolmogorov-Smirnov statistics = 0.01667. Exact permutation p-values for significant genes were computed based on 10 available permutations. Nominal significance level of each univariate test: 0.05. Number of genes significant at 0.005 level of the univariate test: 772

See Supplementary File 1

Supplementary Table S2: Differentially expressed genes among Class 1 (treatment with TNF-a, 6 hours) and Class 2 (parallel untreated control) sorted by p-value of the univariate test. Type of univariate test used: two-sample T-test. Random variance model for univariate test: OFF. Number of genes for random variance estimation: 47,199. Number of genes that passed filtering criteria: 9,022. Exact permutation p-values for significant genes were computed based on 10 available permutations. Nominal significance level of each univariate test: 0.001. Number of genes significant at 0.001 level of the univariate test:106

See Supplementary File 2

Supplementary Table S3: Differentially expressed genes among Class 1 (treatment with PD16+TNF-a, 6 hours) and Class 2 (parallel treatment with TNF-a alone) sorted by p-value of the univariate test. Number of genes for random variance estimation: 47,199. Number of genes that passed filtering criteria: 47,199. Type of univariate test used: two-sample T-test with random variance model. Random variance model parameters: a = 1.53829, b = 76.77471, Kolmogorov-Smirnov statistics = 0.0178. Exact permutation p-values for significant genes were computed based on 10 available permutations. Nominal significance level of each univariate test: 0.005. Number of genes significant at 0.005 level of the univariate test: 2,947

See Supplementary File 3

Supplementary Table S4: Differentially expressed genes among Class 1 (treatment with PD168393+TNF-a, 6 hours) and Class 2 (parallel treatment with TNF-a alone) sorted by p-value of the univariate test. Type of univariate test used: two-sample T-test. Number of genes for random variance estimation: 47,199. Number of genes that passed filtering criteria: 9,022. Random variance model for univariate test: OFF. Exact permutation p-values for significant genes were computed based on 10 available permutations. Nominal significance level of each univariate test: 0.001. Number of genes significant at 0.001 level of the univariate test: 545

See Supplementary File 4

Supplementary Table S5: Primers used for quantitative real-time RT-PCR

Gene	Sense Primer	Antisense Primer
IFN-κ	GGATAGACAATTTCCTGAAAGAAAAG	TCTTGCTTGAAGGTAGATGATTCTT
IRF1	CCCTCCCTTCTTGATATCCCA	CGACTGGCAGCCTGGCT
IRF9	ACCAGGATGCTGCCTTCTT	TCCTGTGTCCCCCTCCTTAT
IFNAR2	CCTTAAAATGCACCCTCCTTC	TTCCTCCTATTTTGGCAGATTC
STAT1	TGAGTTGATTTCTGTGTCTGAAGTT	ACACCTCGTCAAACTCCTCAG
STAT2	CCTGAAGGATCTCTGGAATGA	GGTGCCAGACATGGTCTTCT
IFIT2	TGGTGGCAGAAGAGGAAGAT	GTAGGCTGCTCTCCAAGGAA
CYP1A1	TGGTCAAGGAGCACTACAAAACC	TGATGTCCCGGATGTGGC
CCL2	AACCACAGTTCTACCCCTGGG	TAATGATTCTTGCAAAGACCCTCA
CXCL10	TGGCATTCAAGGAGTACCTCTCT	CTGATGCAGGTACAGCGTACG
IL8	CTCTGTGTGAAGGTGCAGTTT	GGGTGGAAAGGTTTGGAGTAT
TNF-α	CAGCCTCTTCTCCTTCCTGAT	GCCAGAGGGCTGATTAGAGA
β ACTIN	CCTCACCCTGAAGTACCCCA	TCGTCCCAGTTGGTGACGAT
